# *Cannabis sativa* L. Bioactive Compounds and Their Protective Role in Oxidative Stress and Inflammation

**DOI:** 10.3390/antiox11040660

**Published:** 2022-03-29

**Authors:** Dalia M. Kopustinskiene, Ruta Masteikova, Robertas Lazauskas, Jurga Bernatoniene

**Affiliations:** 1 Institute of Pharmaceutical Technologies, Faculty of Pharmacy, Medical Academy, Lithuanian University of Health Sciences, Sukileliu pr. 13, 50161 Kaunas, Lithuania; daliamarija.kopustinskiene@lsmuni.lt; 2 Department of Pharmaceutical Technology, Faculty of Pharmacy, Masaryk University, 61200 Brno, Czech Republic; masteikovar@pharm.muni.cz; 3 Institute of Physiology and Pharmacology, Medical Academy, Lithuanian University of Health Sciences, 50161 Kaunas, Lithuania; robertas.lazauskas@lsmuni.lt; 4 Department of Drug Technology and Social Pharmacy, Faculty of Pharmacy, Medical Academy, Lithuanian University of Health Sciences, Sukileliu pr. 13, 50161 Kaunas, Lithuania

**Keywords:** *Cannabis sativa*, delta-9-tetrahydrocannabinol, cannabidiol, oxidative stress, inflammation

## Abstract

Cannabis (*Cannabis sativa* L.) plants from the family *Cannabidaceae* have been used since ancient times, to produce fibers, oil, and for medicinal purposes. Psychoactive delta-9-tetrahydrocannabinol (THC) and nonpsychoactive cannabidiol (CBD) are the main pharmacologically active compounds of *Cannabis sativa*. These compounds have, for a long time, been under extensive investigation, and their potent antioxidant and inflammatory properties have been reported, although the detailed mechanisms of their actions have not been fully clarified. CB1 receptors are suggested to be responsible for the analgesic effect of THC, while CB2 receptors may account for its immunomodulatory properties. Unlike THC, CBD has a very low affinity for both CB1 and CB2 receptors, and behaves as their negative allosteric modulator. CBD activity, as a CB2 receptor inverse agonist, could be important for CBD anti-inflammatory properties. In this review, we discuss the chemical properties and bioavailability of THC and CBD, their main mechanisms of action, and their role in oxidative stress and inflammation.

## 1. Introduction

**Figure 1:**
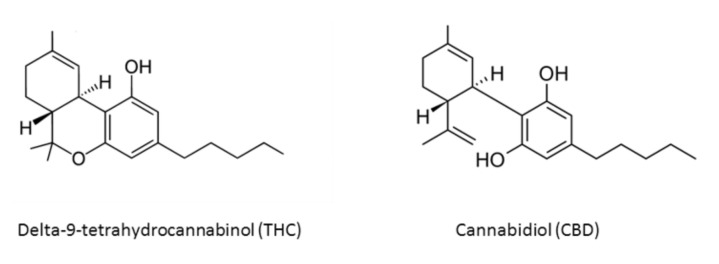
Chemical structures of main *Cannabis sativa* active compounds.

Cannabis (*Cannabis sativa* L.) plants, from the family *Cannabidaceae,* originate from Central Asia, where they were grown to produce fibers, oil, and for medicinal purposes, as probably the oldest psychotropic drug used since ancient times. Archeological discoveries have shown that cannabis has been known in China since around 4000 BC [1]. Cannabis is an annual, dioecious, flowering herb, with characteristic palmate leaves with a venation pattern (Figure 1). There are three known subspecies —*Cannabis sativa* ssp. *sativa* (L.)*, Cannabis sativa* ssp. *indica* (Lam.), and *Cannabis sativa* ssp. *ruderalis* (Janisch), although, in some studies, these plants are classified as distinct species [2,3,4]. *Cannabis sativa* is the most widely spread variety, growing in both tropical and temperate climates. The two main preparations derived from cannabis are marijuana and hashish. The name marijuana originates from Mexica, where it was used to describe cheap tobacco. Today, marijuana is the name for the dried leaves and flowers of the cannabis plant. Hashish is the Arabic name for Indian hemp, now used for the viscous resin of the plant [1].

The Emperor of China, Shen Nung, first described the properties and therapeutic uses of cannabis in his book of Chinese medicinal herbs, written in 2737 BC [5,6]. Soon afterwards, the plant was cultivated for its fibers, seeds, recreational consumption, and use in medicine. It then spread to India from China [5,6]. In 1839, William O’Shaughnessy, who worked in India, described the analgesic, appetite stimulant, antiemetic, muscle relaxant, and anticonvulsant properties of cannabis, and, thus, the medical use of cannabis began [1,5,6]. In 1854, cannabis was included in the United States Dispensatory [7], and was freely available in pharmacies in western countries; it was also included in the British Pharmacopoeia, as an extract and tincture, for over 100 years [6]. After the implementation of the Marihuana Tax Act in 1937, it became impossible to prescribe any preparation containing cannabis in the US, and, in 1942, cannabis was removed from the United States Pharmacopoeia [1,5,6]. The ban on cannabis was introduced by Great Britain and most European countries after adopting the Convention on Psychotropic Substances, issued by the United Nations in 1971 [1]. Since then, many studies have reported beneficial effects of cannabis use for various chronic and debilitating disorders, such as cancer, Alzheimer’s disease, and AIDS [1,5]. Therefore, currently, there are ongoing debates about the legalization of *Cannabis sativa* use for medical and recreational purposes in many countries [8].

The aim of this narrative review is to provide an overview of the chemical properties and bioavailability of THC and CBD, and their main mechanisms of action, with a focus on their role in oxidative stress and inflammation. The summary figures in this review were prepared with the aid of Serif DrawPlus X8 (Serif (Europe) Ltd., Nottingham, United Kingdom) and MS PowerPoint programs.

## 2. Chemical Properties of *Cannabis sativa* Bioactive Compounds

More than 538 known chemical compounds are present in cannabis, around 100 of which are classified as cannabinoids, which are aryl-substituted meroterpenes [8,9]. There are also eighteen different chemical classes of substances, such as nitrogen compounds, amino acids, hydrocarbons, carbohydrates, terpenes, organics, and fatty acids [8,10]. The most important active compounds in cannabis are the psychoactive cannabinoid [11,12] delta-9-tetrahydrocannabinol (THC) [13,14], due to its lipophilic structure, enabling it to cross the blood–brain barrier, and nonpsychoactive cannabidiol (CBD) [15,16] (Figure 1).

The highest amount of THC is found in female inflorescences of cannabis [17,18]. Depending on the THC content, the following three types of cannabis are defined: drug type, with a high THC/CBD ratio (above 1), which is psychoactive (chemotype I), used to make drugs such as marijuana and hashish; medium type (chemotype II), with a medium THC/CBD ratio (close to 1), which is nonpsychoactive or has a low activity; fiber type (chemotype III), called hemp, which has <0.3 percent THC and is characterized by a low THC/CBD ratio (below 1)—it is nonpsychoactive, and is used to make fiber and edible oil [9,19]. The cannabinoids of fiber-type cannabis are mainly cannabinoid acids, as follows: cannabidiolic acid, cannabigerolic acid, and their decarboxylated derivatives—cannabidiol and cannabigerol. Cannabichromenic acid, cannabichromene, and THC degradation products—cannabinolic acid and cannabinol—are found in lesser amounts in hemp [1,9,19]. The THC content of industrial hemp preparations is limited to not exceed 0.2%.

## 3. Bioavailability of *Cannabis sativa* Main Active Compounds

Following administration of THC or CBD by inhalation, the peak plasma concentrations are reached rapidly, within 3 to 10 min, and remain higher than after oral administration of cannabinoids [20,21]. The mean systemic bioavailability after inhalation is 10–30% for THC and about 31% for CBD [20,21].

Inhalation or oral absorption reduces the first-pass metabolism of cannabinoids. Both THC and CBD are highly lipophilic substances with low oral bioavailability (only about 6%). In addition, the peak oral plasma concentrations are reached in approximately 120 min, resulting in a higher dose of THC or CBD oral formulations, requiring long-term systemic exposure [20,21].

Transdermal administration of THC or CBD helps to prevent first-pass metabolism, but the lipophilicity of cannabinoids complicates the ability of the substance to penetrate through the skin [20,21]. In vitro studies of CBD penetration through human skin have shown that the permeability of CBD through the skin is about 10 times higher than THC, as CBD is a less lipophilic substance than THC [22]. The bioavailability of THC after the administration of rectal suppositories was about 13.5% [23,24]; furthermore, THC did not accumulate in the blood of patients at a daily dose of 10–15 mg [25]. Following the rectal administration of 2.5–5 mg of active substance, the peak plasma concentrations ranged from 1.1 to 4.1 ng/mL over 2 to 8 h [21,25]. When administered rectally, the bioavailability was approximately two-fold higher than when administered orally, due to higher absorption and lower first-pass metabolism [21,25].

Cannabinoids are rapidly distributed in tissues with a developed vascular system (e.g., lung, heart, liver, and brain), depending on body weight and structure [20,21]. After prolonged use, cannabinoids may accumulate in adipose tissue. The volume of distribution (Vd) of CBD and THC is high, at 32 L/kg after intravenous administration and 3.4 L/kg after inhalation [20,21]. 

Both THC and CBD are metabolized in the liver. The most important enzymes for THC metabolism are cytochrome P450 (CYP 450) and isozymes CYP2C9, CYP2C19, and CYP3A4. THC is converted to 11-hydroxyTHC and 11-carboxy-THC, and subsequently undergoes glucuronidation [20,21]. Excretion of THC is mainly via the feces and urine. Other tissues that express CYP450—the brain and small intestine—can also metabolize THC [20,21]. Due to its lipophilic properties, THC can cross the placenta, and can be excreted in human breast milk [20,21]. CBD is metabolized in the liver, and the major enzymes involved in this process are CYP2C19 and CYP3A4, and, in addition, CYP1A1, CYP1A2, CYP2C9, and CYP2D6 [20,21]. After hydroxylation to 7-hydroxycannabidiol (7-OH-CBD), the products formed are excreted in the gut during further metabolization, or a lesser amount of the metabolites may be excreted in the urine [20,21]. The activity of CBD metabolites in humans has not been extensively investigated yet.

A fast initial half-life of about 6 min and long terminal half-life of about 22 h, related to accumulation of the substance in lipid-rich tissues, have been reported for THC [20,21]. The elimination half-life of CBD is long, approximately 24 ± 6 h after intravenous administration or 31 ± 4 h after inhalation [20,21]. After prolonged administration of CBD, the elimination half-life is 2 to 5 days [20,21].

THC can aggravate psychotic disorders [26,27], and its chronic use can cause depression, anxiety, and decreased motivation [28,29]. Furthermore, THC can cause an acute increase in blood pressure and heart rate in a dose-dependent manner [30]. In contrast to THC, CBD is well tolerated and has relatively few serious adverse effects [31]; however, drug–drug interactions, diarrhea, fatigue, vomiting, somnolence, and hepatic abnormalities have been reported in several studies [32,33]. Due to adverse reactions, cannabinoid therapy should not be used for patients with severe psychiatric, cardiac, renal, or hepatic disorders [20,21].

## 4. Main Mechanisms of Action of *Cannabis sativa* Bioactive Compounds

**Figure 2:**
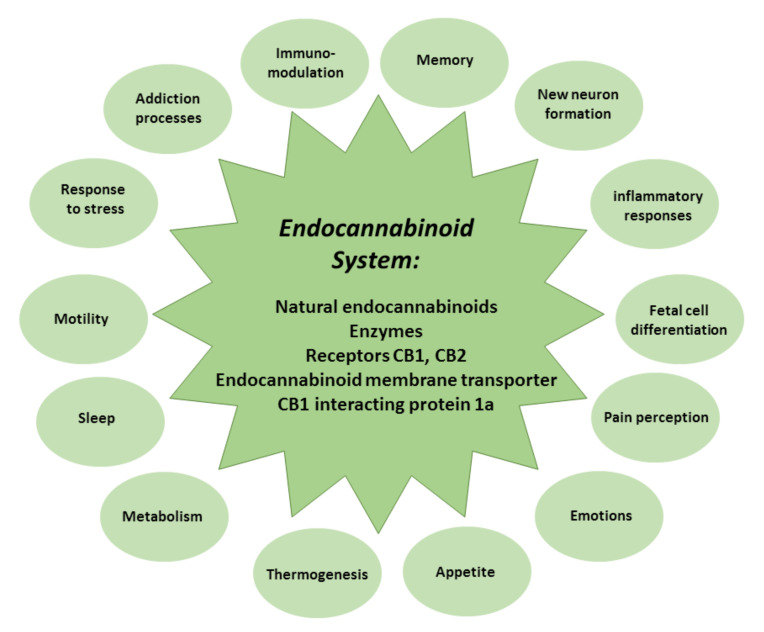
The composition and role of the endocannabinoid system.

During extensive studies on the effects of THC, a receptor for THC in the central nervous system (now known as the CB1 receptor) was cloned in 1990 [34], and, in 1992, a major natural ligand of CB1 was identified and named anandamide [35]. Another cannabinoid receptor, the peripheral or CB2 receptor, was cloned from macrophages and the spleen in 1993 [36]. Later, other components of the endocannabinoid signaling system were discovered [9,37] (Figure 2).

The endocannabinoid system comprises natural endocannabinoids—happiness molecules—N-arachidonoylethanolamine (anandamide) and 2-arachidonoylglycerol, the enzymes that participate in their synthesis and metabolism, CB1 and CB2 receptors, endocannabinoid membrane transporters, and CB1 interacting protein 1a, capable of controlling CB1 receptor signal transduction [37,38]. The endocannabinoid system modulates memory, new neuron formation, immune and inflammatory responses, and fetal cell differentiation, and regulates pain strength, emotions, appetite, thermogenesis, metabolism, sleep, motility, response to stress, and addiction processes [39] (Figure 2).

The cannabinoid receptors CB1 and CB2 are members of the G-protein-coupled receptor (GPCR) family [38]. They regulate important intracellular signal transduction pathways, comprising activation of the phosphorylation of mitogen-activated protein kinases (MAPK) and A-type potassium channels, and suppression of adenylyl cyclase activity, D-type potassium, and calcium channels [38]. CB1 receptors are widespread in neuron terminals throughout the nervous system, mainly in the brain, but also in the spinal cord and peripheral sensory nerve endings [12,40,41]. In the central nervous system, CB1 receptors in the amygdala, thalamus, and midbrain periaqueductal grey matter modulate nociception. The abundance of CB1 receptors in other regions of the brain accounts for the other effects of cannabinoids. For example, CB1 receptors in the basal ganglia, cerebellum, and hypothalamus modulate motor activity, motor coordination, and appetite and sedation, respectively [7]. The stimulation of CB1 receptors reduces neuronal excitability and the release of the neurotransmitters c-aminobutyric acid and glutamate in the cortical, limbic, and other regions involved in nociception [12,40,41].

In contrast, CB2 receptors are mainly found in immune tissues (e.g., spleen and tonsils) and immune cells (e.g., monocytes, and B and T cells), although some are also located in the brain. The stimulation of peripheral CB2 receptors results in anti-inflammatory and immunomodulating effects, and, thus, plays a role in alleviating inflammatory types of pain, as well as neuropathic pain [42,43]. CB2 receptors are also important in bone remodeling [44] and atherosclerosis [45].

**Figure 3:**
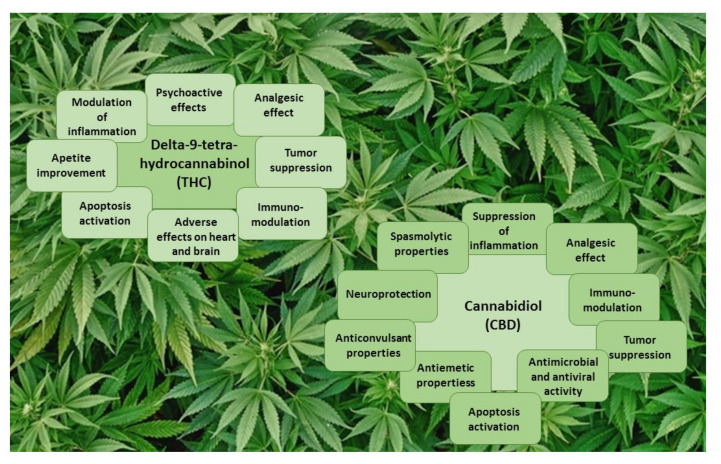
The main effects of THC and CBD.

THC is a potent partial agonist that binds with high affinity to both the CB1 and CB2 cannabinoid receptors [46], with dissociation constants (K_i_) of 10 and 24 nM, respectively [47]. The main psychoactive effects of THC, as well as its analgesic effect, are mediated by CB1 receptors [47] (Figure 3). CB2 receptors are responsible for the immunomodulatory properties of THC [38,47]. In addition, THC can act as an agonist of G-protein-coupled receptors (GPR55 and GPR18), the peroxisome proliferator-activated receptor (PPARγ), and transient receptor potential channels (TRPA1, TRPV2, TRPV3, and TRPV4), and as an antagonist of transient receptor potential channel TRPM8 and 5-HT3 receptor A, and can increase anandamide and adenosine levels [38,48].

Numerous studies have shown that CBD possesses analgesic [16,49], neuroprotective [40], anticonvulsant [11], antiemetic [50], spasmolytic [51], and anti-inflammatory [11,12,52] properties (Figure 3). Unlike THC, CBD has a very low affinity for both CB1 and CB2 receptors, with K_i_ of 4359 and 2860 nM, respectively [47]. CBD is a potent antagonist of CB1 and CB2 receptor agonists [38,46,47]. The action of CBD, as a CB2 receptor inverse agonist, may be responsible for its anti-inflammatory properties [38]. CBD behaves as a negative allosteric modulator of both CB receptors [53,54]. CBD could act as an agonist of transient receptor potential channels (TRPA1, TRPV1, TRPV2, and TRPV3), the peroxisome proliferator-activated receptor (PPARγ), 5-HT1A (serotonine 1A) receptor, and adenosine A1 and A2 receptors, and as an antagonist of G-protein-coupled receptors (GPR55 and GPR18) and 5-HT3 receptor A [38,55,56,57]. CBD is also an inverse agonist of G-protein-coupled receptors (GPR3, GPR6, and GPR12) and elevates anandamide levels [55,56,57].

## 5. Effects of *Cannabis sativa* Bioactive Compounds on Oxidative Stress and Inflammation

**Figure 4:**
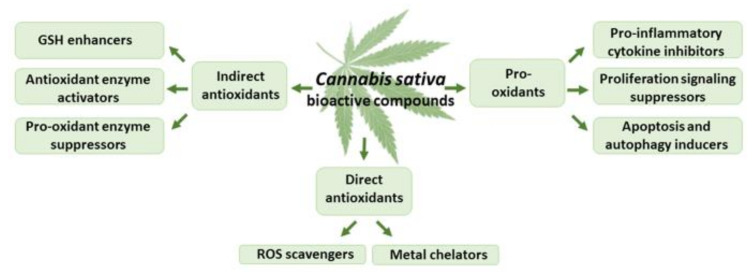
The effects of THC and CBD on oxidative stress.

Both THC and CBD exhibit antioxidant activity comparable to that of vitamins E and C, being capable of scavenging free radicals, reducing metal ions, and protecting oxidation processes [58,59] (Figure 4). The phenolic groups readily oxidized to quinoid forms [60] and unsaturated bonds found in non-olivetolic fragments of THC and CBD could be responsible for their antioxidant properties [58]. THC and CBD protected rat neuronal cell cultures against hydroperoxide-induced oxidative damage (EC_50_ of 2–4 μM) at a degree comparable to that of ascorbate and tocopherol [40]. Moreover, both cannabinoids were effective as direct antioxidants, protecting rat cortical neuron cultures against the damage of toxic levels of the neurotransmitter glutamate [61].

THC and CBD, at submicromolar concentrations, prevented the oxidative cell death of B lymphoblastoid cells and fibroblasts in serum-deprived medium, via direct antioxidant action [62]. CBD, as a direct antioxidant, provided protection against brain injuries at a dose of 2 mL i.p. bolus, which supplied either 20 or 40 mg/kg CBD, in the rat models of ethanol-induced neurotoxicity [63], and at a dose of 3 mg/kg for two weeks of 6-hydroxydopamine-induced neurotoxicity [64] and Parkinson’s disease [65]. A *Cannabis sativa* extract rich in THC and CBD, at a dose of 15 or 30 mg/kg for 8 days, provided protection against oxidative damage, due to its antioxidant activity, thus alleviating diabetic neuropathic pain in streptozotocin-induced diabetic rats [66]. THC and CBD provided protection against oxidative neuronal cell death in the mouse hippocampal HT22 cell line and rat primary cerebellar cell culture models, proving that CB1 has not been involved in these neuroprotective effects [67]. THC and CBD, at logarithmic concentrations of 0.1, 1, 10, and 100 μM, increased insulin release, and Pdx1, Glut2, and thiol molecule expression, due to the significant reduction in ROS, while the oxidative stress parameters were decreased in the aged pancreatic islets [68]. In addition, THC (3 µM) provided protection against N-methyl-D-aspartate-induced apoptosis in AF5 cells, by blocking ROS generation [69].

Cannabinoids also act as indirect antioxidants, capable of modulating the redox balance via regulation of the GSH level, activation of antioxidant enzymes, and suppression of pro-oxidant enzymes [70,71,72,73] (Figure 4). CBD, 1, 10, or 20 mg/kg i.p. for 8 weeks, increased the mRNA level, as well as the activity of superoxide dismutase, in the mouse model of diabetic cardiomyopathy type I and in human cardiomyocytes treated with 3-nitropropionic acid or streptozotocin [74]. In neuropathic and inflammatory pain models in rats, CBD (2.5–20 mg/kg for a week) decreased lipid peroxide and nitric oxide levels and modulated the activity of glutathione-related enzymes [75]. CBD (120 mg/kg of body weight; 2.5% *w/w* in petrolatum, applied topically (20 min) every 12 h for 4 weeks) increased the levels of glutathione, and suppressed the activities of phospholipase A2 and cyclooxygenases in the skin of nude rats chronically irradiated with UVA/UVB [76]. CBD (injected at 50, 100, and 200 ng/rat for five consecutive days) reduced the infarction volume and malondialdehyde level in cortical and striatal areas of the rat brain, and elevated the activity of superoxide dismutase and catalase enzymes in the cortex and striatum [77]. 

**Figure 5:**
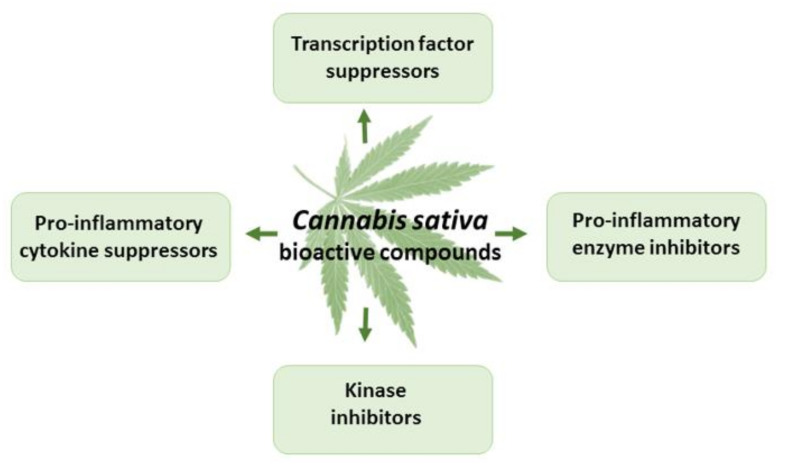
The effects of THC and CBD in inflammation.

Cannabinoids have demonstrated strong anti-inflammatory properties in numerous in vitro and in vivo studies [1,8,70,71] (Figure 5). THC exerts anti-inflammatory actions, mainly due to CB2 receptor activation, suppressed cytokine production, inhibition of Th-1 cells, activation of Th-2 cells, induction of apoptosis, and suppression of cell proliferation [78,79,80]. The anti-inflammatory properties of CBD are due to interactions with CB1, CB2, the CB2/5HT1A complex, TRPV1, and adenosine receptors, as well as the activation of GPR55 and peroxisome proliferator-activated receptor gamma, and the inhibition of fatty acid amide hydrolase [56,80,81]. CBD suppresses pro-inflammatory cytokines, such as IL-1α, IL-1β, IL-6, and tissue necrosis factor α (TNF-α), in pre-clinical in vitro and in vivo models of inflammation and cancer [81].

THC diminished the levels of the following pro-inflammatory cytokines: tumor necrosis factor alpha (TNF-α), granulocyte-macrophage colony-stimulating factor, and interferon-c cytokine [82]. THC also inhibited lipopolysaccharide-induced mRNA expression for interleukins IL-1a, IL-1b, IL-6, and TNF-α in a rat microglia culture [83]. THC (15 mg/kg/d) suppressed the mammalian target of rapamycin complex 1, activated apoptosis and autophagy [84], suppressed cell proliferation [85], down-regulated vascular endothelial growth factor signaling, and inhibited metalloproteinase 2 [86,87].

CBD is able to activate the PPAR-c receptor, thus mediating important anti-inflammatory and antioxidant effects in Parkinson’s disease models [88]. Many studies suggest that the activation of both cannabinoid receptors, CB1 and CB2, alleviates intestinal inflammation in a variety of mouse colitis models [89]. Cannabinoids reduce the hypersensitivity of internal organs and abdominal pain, as well as intestinal peristalsis and diarrhea, associated with colitis [90,91,92,93]. In addition, CB1 receptors inhibit secretory processes and also modulate the barrier functions of the intestinal epithelium. Thus, the endocannabinoid system is a promising target in the treatment of inflammatory bowel diseases [89]. Due to their complex anti-inflammatory effects, cannabinoids are effective in inhibiting the development of colitis [89]. CBD was effective in reducing intestinal inflammation in a CD1 mouse model, when intestinal inflammation was induced by trinitrobenzene sulfonic acid and was treated with CBD, either administered orally (10 mg/kg) or rectally (20 mg/kg) [94]. The study concluded that rectal administration of CBD preparations is also effective in the treatment of intestinal inflammation [94]. The efficacy and tolerability of CBD-containing oral cannabis extract capsules have been studied in patients with ulcerative colitis, and the patients’ conditions have been shown to improve [95]. Thus, due to its anti-inflammatory and analgesic effects, CBD can be used for the topical treatment of inflammatory bowel disease [89,93].

Gut signaling can influence brain function, and recent research revealed that the gut–brain axis may play a key role in the common link between gastrointestinal and neurological diseases [96,97]. In the murine model of multiple sclerosis—an experimental autoimmune encephalomyelitis—C57BL/6 mice were treated with an i.p. injection of 10 mg/kg each of THC + CBD (1:1 ratio) or a vehicle (2% dimethylsulfoxide, DMSO; 20% ethanol diluted in PBS) daily, for 10 days following induction of the disease [98]. The treatment reduced mucin-degrading bacterial species, such as *Akkermansia muciniphila*, reduced disease symptoms, and caused a significant decrease in inflammatory cytokines [98]. However, clinical research on the role of THC and CBD in modulating human microbiota is still limited, but several studies have demonstrated the importance of the endocannabinoid system in the regulation of gut microbiota, the gut–brain axis, inflammatory diseases, and obesity [96,97,99,100,101,102,103,104]. Changes in intestinal permeability and disruption of the intestinal microbiota are responsible for the inflammation processes in obesity [105]. Lipopolysaccharides from the intestinal microbiota can trigger chronic inflammation, leading to insulin resistance, through activation of Toll-like receptor 4 [106]. The triple interactions between the gut microbiota, host immune system, and metabolism are important factors in obesity and diabetes [101,107]. Cellular energy turnover, insulin resistance, fat deposition, and inflammation are affected by obesity-related microbiota [108]. In addition, the microbiota in the gut can influence metabolism, adiposity, homoeostasis, and energy balance, as well as appetite regulation [108]. Furthermore, some bacteria strains and their metabolites may directly alter vagal stimulation, thus affecting the brain, or indirectly regulate brain activity via immune-neuroendocrine processes [108]. The interplay between the microbiome gut–brain axis and the endocannabinoid system is very important in the development of Alzheimer’s disease and other neurodegenerative disorders that have recently been linked to dysbiosis [100,109]. Moreover, the interactions between the gut microbiota and the immune system can be perceived through regulation by the endocannabinoid system [109]; thus, the modulators of this system could be the potential precursors of drugs or a part of alternative complimentary therapy for the treatment of metabolic and neurodegenerative diseases.

## 6. Conclusions and Future Perspectives

Cannabinoids exhibit interesting therapeutic potential as antiemetics, appetite stimulants in debilitating diseases (cancer and AIDS), analgesics, and anti-inflammatory remedies in the treatment of multiple sclerosis, spinal cord injuries, Tourette’s syndrome, epilepsy, and glaucoma [1,39]. Further well-controlled trials are needed to elucidate the potential of cannabinoids in clinical practice.

Cannabinoids might be prospective future drugs in the treatment of cancer-related chronic pain conditions and inflammation. They could even replace opiates, which are highly addictive and have much more serious side effects.
